# Comparing the Effect of Dexmedetomidine and Midazolam in Patients with Brain Injury

**DOI:** 10.3390/brainsci12060752

**Published:** 2022-06-08

**Authors:** Yanxia Huang, Yunxin Deng, Renjing Zhang, Mei Meng, Dechang Chen

**Affiliations:** Department of Critical Care Medicine, Ruijin Hospital, Shanghai Jiao Tong University School of Medicine, Shanghai 201801, China; hyx12805@rjh.com.cn (Y.H.); yoyo_ssmu@163.com (Y.D.); zrj12781@rjh.com.cn (R.Z.); mengmeng74@163.com (M.M.)

**Keywords:** dexmedetomidine, midazolam, qEEG, post-craniotomy, sedative

## Abstract

Background: Studies have shown that dexmedetomidine improves neurological function. Whether dexmedetomidine reduces mortality or improves quantitative electroencephalography (qEEG) among patients post-craniotomy remains unclear. Methods: This single-center randomized study was conducted prospectively from 1 January 2019 to 31 December 2020. Patients who were transferred to the ICU after craniotomy within 24 h were included. The analgesic was titrated to a Critical care Pain Observation Tool (CPOT) score ≤2, and the sedative was titrated to a Richmond Agitation–Sedation Scale (RASS) score ≤−3 for at least 24 h. The qEEG signals were collected by four electrodes (F3, T3, F4, and T4 according to the international 10/20 EEG electrode practice). The primary outcome was 28-day mortality and qEEG results on day 1 and day 3 after sedation. Results: One hundred and fifty-one patients were enrolled in this study, of whom 77 were in the dexmedetomidine group and 74 in the midazolam group. No significant difference was found between the two groups in mortality at 28 days (14.3% vs. 24.3%; *p* = 0.117) as well as in the theta/beta ratio (TBR), the delta/alpha ratio (DAR), and the (delta + theta)/(alpha + beta) ratio (DTABR) between the two groups on day 1 or day 3. However, both the TBR and the DTABR were significantly increased in the dexmedetomidine group. The DTABR in the midazolam group was significantly increased. The DAR was significantly increased on the right side in the dexmedetomidine group (20.4 (11.6–43.3) vs. 35.1 (16.7–65.0), *p* = 0.006) as well as on both sides in the midazolam group (Left: 19.5 (10.1–35.8) vs. 37.3 (19.3–75.7), *p* = 0.006; Right: 18.9 (10.1–52.3) vs. 39.8 (17.5–99.9), *p* = 0.002). Conclusion: Compared with midazolam, dexmedetomidine did not lead to a lower 28-day mortality or better qEEG results in brain injury patients after a craniotomy.

## 1. Introduction

Analgesics and sedatives are important and common therapies for patients after craniotomies caused by brain traumas, cerebral hemorrhages, and cerebral infarctions [1,2,3]. Analgesics and sedatives can be used to control intracranial pressure (ICP), prevent agitation, bucking, or strain against tubes [3]. The appropriate depth of analgesia and sedation can also improve the coupling of regional blood flow to metabolic demands, result in a higher brain oxygenation with a lower cerebral blood flow, and decreased ICP [4].

Midazolam, as a first-line sedative, is commonly used in clinical practice [5]. As a highly selective central agonist of the α2 adrenergic receptor, dexmedetomidine has been administered in many therapeutic procedures safely and efficiently [6,7,8]. Animal studies showed that dexmedetomidine reduced neuronal death by protecting against neural autophagy and neuroinflammation, enhanced cognitive and motor recovery following traumatic brain injury, and alleviated cerebral ischemic reperfusion injury [9,10,11]. Hence, dexmedetomidine was recommended in patients after craniotomy in recent years [12,13,14,15,16]. However, the efficacy of dexmedetomidine on patients post-craniotomy was unknown.

Electroencephalography (EEG) is the most used neuroimaging technique in an intensive care unit (ICU) because it is non-invasive, portable, and inexpensive [17]. It can serve as a convenient bedside assessment tool for patients in an ICU. Quantitative electroencephalography (qEEG) can provide the objective and real-time quantification of EEG reactivity [18]. Studies found that qEEG metrics correlate with a patient’s outcome [19,20]. Delta/alpha ratio (DAR) and (delta + theta)/(alpha + beta) ratio (DTABR) were reported to be independent predictors for clinical outcome [19]. A higher DAR and a higher DTABR were associated with high mortality.

In the current study, dexmedetomidine and midazolam were investigated to find whether they had a different efficacy on patients post-craniotomy. Our hypothesis is that dexmedetomidine leads to a lower mortality and a lower DAR and DTABR in patients with brain injuries.

## 2. Methods

### 2.1. Ethics

This study was approved by the Ethics Committee of Ruijin Hospital North. This study was registered on the Clinical Trial website (ChiCTR1800016434). Written informed consent was obtained from each participant’s guardian prior to their enrollment in this study.

### 2.2. Study Design

This single-center study was conducted prospectively at the Department of Critical Care Medicine, Ruijin Hospital North Affiliated to the Shanghai Jiaotong University School of Medicine from 1 January 2019 to 31 December 2020.

### 2.3. Patients

1. Inclusion criteria: (1) patients transferred to the ICU after craniotomy within 24 h; (2) patients aged ≥18 years old. 2. Exclusion criteria: (1) sedation duration < 24 h; (2) take benzodiazepines for a long time before this study (more than 3 months); (3) pregnancy; (4) GCS score was 3 and electrocerebral silence; (5) serious hepatic dysfunction (Child–Pugh class B or C); (6) serious renal dysfunction (undergoing dialysis before surgery; or serum creatinine > 445 mmol/L and/or BUN > 20 mmol/L in preoperative laboratory examination); (7) death occurring within 24 h after surgery; (8) craniotomy for brain tumor in this period; (9) refusal to sign informed consent.

### 2.4. Randomization and Study Regimen

Patients were screened within 3 h after admission to ICU. Patients who met inclusion criteria were enrolled in this study. The random tables were developed in Excel sheets prior to the study. Patients enrolled one by one according to the order of inclusion. Patients received midazolam (Mid group) or dexmedetomidine (Dex group) for sedative. In both groups, patients received sufentanil or renifentanil for analgesia. All patients underwent analgesia and sedation titration. The analgesic was titrated to Critical care Pain Observation Tool (CPOT) score ≤ 2 and the sedative was titrated to Richmond Agitation–Sedation Scale (RASS) score ≤ −3. Analgesia and sedation were maintained for at least 24 h. If sedation target could not be reached, propofol could be used as a salvage therapy. If seizures occurred, midazolam could be given 2.5 mg to 5 mg, followed by valproate.

### 2.5. Data Collection

The qEEG was recorded within 24 h (day 1) and 48 to 72 h (day 3) after randomization. qEEG was recorded for 4 h each time. Patients’ individual demographic characteristics (e.g., sex, age, body weight, height, and Body mass index (BMI)), diagnosis, pre-existing condition, Acute Physiology and Chronic Health Evaluation (APACHE) II score, and Glasgow Coma score (GCS) before sedative. The complications within 28 days and the 28-day outcome were collected.

**Figure 1:**
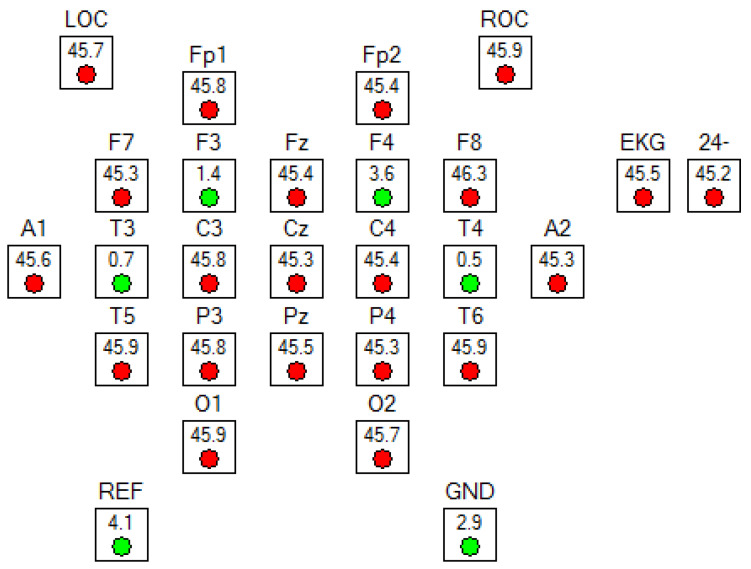
Electroencephalography electrode setup. Four conventional EEG recording sites were used in accordance with the international 10/20 system.

EEG was recorded using the Nicolet range of multimedia EEG systems from standard 4 electrodes (F3, T3, F4, and T4 according to the international 10/20 EEG electrode practice). The electrodes’ impedance is set to be below 5 kOhm. A reference electrode was symmetrically placed over the sagittal midline at FCz to avoid biased electrical potentials towards one hemisphere. The ground electrode was located at Fpz (Figure 1). The EEG data were recording for at least 4 h. Fast Fourier transformation (averaged windows of 5 s with 50% overlap) was used to calculate amplitude (μV) for the EEG bands delta (1–4 Hz), theta (4–8 Hz), alpha (8–13 Hz), and beta (13–30 Hz). The collected information included: EEG amplitude (μV), Coefficient of variation of EEG amplitude (CV), spikes, Alpha variance, percentage of every band, theta/beta ratio (TBR), delta/alpha ratio (DAR), (delta + theta)/(alpha + beta) ratio (DTABR), and spectral entropy.

The primary outcome was 28-day mortality, and qEEG results on day 1 and day 3 after sedation launching. The second outcome was the length of ICU stay and the length of hospital stay.

### 2.6. Statistical Analysis

All statistical analyses were processed with the Statistical Package for Social Sciences 23.0 (SPSS, Inc., Chicago, IL, USA). The categorical variables were presented as number (*n*) and percentage (%). The continuous variables were presented as mean ± standard deviation (SD) or median (25–75% interquartile range, IQR).The unpaired Student’s *t*-test and unpaired Wilcoxon rank-sum test were assessed to analyze continuous variables. The paired Student’s *t*-test was assigned to compare the in-group continuous variables. The chi-squared (χ^2^) test was used to compare categorical variables. A two-sided *p* value ≤ 0.05 was considered statistically significant.

## 3. Results

### 3.1. Characteristics

**Figure 2:**
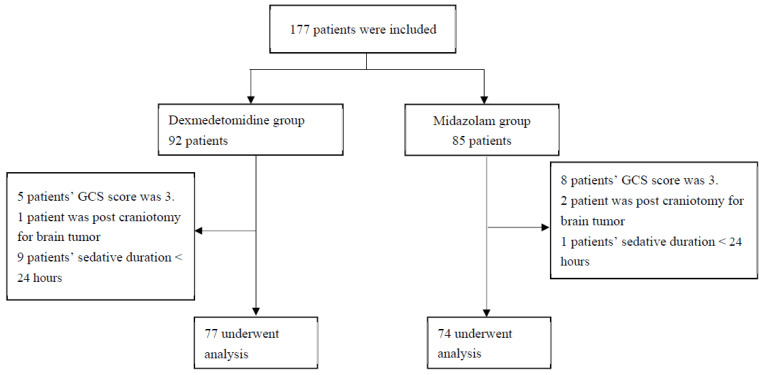
Flowchart of enrollment in the study. GCS: Glasgow Coma score.

From 1 January 2019 through 31 December 2020, we identified 177 eligible patients, of whom 92 patients were assigned to receive dexmedetomidine and the other 85 patients to receive midazolam. In the Dex group, five patients’ GCS scores were three with electrocerebral silence, one patient was post-craniotomy for a brain tumor, and nine patients’ sedative duration was less than 24 h. In the Mid group, eight patients’ GCS scores were three with electrocerebral silence, two patients were post-craniotomy for a brain tumor, and one patients’ sedative duration was less than 24 h. Thus, the trial included 151 enrolled patients, of whom 77 were in the Dex group and 74 were in the Mid group (Figure 2).

The characteristics of patients at baseline were similar in the two groups (Table 1). The mean (±SD) age of the patients was 52.2 ± 13.5 years in the Dex group and 51.8 ± 12.8 years in the Mid group. The percentages of male patients were 67.5% and 70.3%, respectively. There were 33.8% patients in the Dex group that suffered from brain traumas, 64.9% patients suffered from intracerebral hemorrhages, and 1.3% patients suffered from cerebral infarctions. The percentages of attributed illness of patients in the Mid group were 41.9%, 58.1%, and 0, respectively. There was no significant difference between the two groups (*p* = 0.385). The APACHE II scores and GCS scores were 15.2 ± 5.7 versus 15.7 ± 4.7 (*p* = 0.513) and 6.5 ± 3.1 versus 5.7 ± 2.3 (*p* = 0.055), respectively. The RASS scores were similar in both groups (−3.8 ± 0.5 and −3.9 ± 0.4, respectively, *p* = 0.125). The CPOT score was similar between the two groups. The percentage of propofol prescribing was higher in the Dex group, but the difference was not significant. There were no significant differences about the percentage of mechanical ventilations, vasoactive drug dosages, or chronic diseases (Table 1).

### 3.2. Outcomes

There was no significant difference between the two groups in mortality at 28 days. Eleven patients died in the Dex group (14.3%; 95% confidence interval (CI), 6.3 to 22.3) and 18 died in the Mid group (24.3%; 95% confidence interval (CI), 14.3 to 34.3; *p* = 0.117).

The length of the ICU stay was 9.5 ± 7.7 days in the Dex group and 11.9 ± 11.6 days in the Mid group (*p* = 0.133). The length of the hospital stay was 23.5 ± 19.8 days in the Dex group and 25.3 ± 18.2 days in the Mid group (*p* = 0.562). The complications within 28 days between the two groups were similar (*p* = 0.450). A total of 79.2% of patients in the Dex group developed pneumonia, 1.3% of patients developed intracranial infections, 2.6% of patients developed bloodstream infections, 1.3% of patients developed cerebral hemorrhages, 0% of patient developed cerebral infarctions, 1.3% of patients developed acute kidney injuries, and 1.3% of patients developed ventricular tachycardia. The incidence was 91.9%, 4.1%, 1.4%, 4.1%, 4.1%, 0%, and 1.4% in the Mid group, respectively (Table 2).

There were 58 patients and 57 patients that recorded qEEGs on day 1 in the Dex and Mid groups, respectively. Subsequently, 46 and 42 patients recorded qEEGs on day 3, among whom were in the dexmedetomidine and midazolam groups.

On the first day, there was no significant difference in EEG amplitude, CV, spikes, Alpha variance, percentage of delta, theta, alpha, and beta bands on the left, percentage of delta, alpha, and beta bands on the right, and spectral entropy (Table 3). The theta band on the right in the Dex group was higher than that in the Mid group (7.6 ± 5.1 vs. 5.9 ± 3.9, respectively; *p* = 0.045). On the third day, there was no significant difference in EEG amplitude, CV, spikes, Alpha variance, percentage of delta, alpha, and beta bands on the left, percentage of delta, alpha, and beta bands on the right, and spectral entropy (Table 4). The theta bands on both sides in the Dex group were higher than that in the Mid group (Left: 8.4 (3.8–15.0) vs. 5.0 (3.1–10.9), respectively; *p* = 0.018 and Right: 8.5 ± 4.7 vs. 6.2 ± 4.2, respectively; *p* = 0.021).

There was no significant difference in the theta/beta ratio (TBR), the delta/alpha ratio (DAR), and the (delta + theta)/(alpha + beta) ratio (DTABR) between the two groups on day 1 and day 3 (Table 5). But both the TBR and the DTABR were significantly increased in the Dex group. The DTABR in the Mid group was significantly increased. The DAR in the Dex group was significantly increased on the right (20.4 (11.6–43.3) vs. 35.1 (16.7–65.0), *p* = 0.006). But the DAR in the Mid group was significantly increased on both sides (Left: 19.5(10.1–35.8) vs. 37.3(19.3–75.7), *p* = 0.006; Right: 18.9 (10.1–52.3) vs. 39.8 (17.5–99.9), *p* = 0.002).

### 3.3. Discussion

In this study, the administration of dexmedetomidine did not result in a lower 28-day mortality than the midazolam group among patients post-craniotomy for brain trauma, cerebral hemorrhage, or cerebral infarction. The length of the ICU stay and the length of the hospital stay in the Dex group were shorter than that in the Mid group, but the differences were not significant. That might be due to the small sample size of this study.

Dexmedetomidine is a highly selective α2-adrenoreceptor agonist with sedative, analgesic, and sympatholytic properties. Dexmedetomidine was found to have a significant neuroprotective effect in an in vitro model for traumatic brain injury [21]. Previous studies found that when the concentration of dexmedetomidine was 0.1 μmol/L, the protective effort on traumatically injured hippocampal cells had a maximum effect. In our study, dexmedetomidine was used for sedation, and the concentration of dexmedetomidine in serum or cerebrospinal fluid was not available in our institution. If we test the concentration of dexmedetomidine and reached the 0.1 μmol/L of dexmedetomidine in cerebrospinal fluid, the patients’ outcome may be improved.

Several studies have shown that dexmedetomidine improves neurological function after traumatic brain injury through a variety of mechanisms. In Zhao’s study, they found that post-synaptic density 95 (PSD95) formed a complex with the N-methyl-D-aspartic acid (NMDA) receptor subunit (NR2B) and neuronal nitric oxide synthase (nNOS), inducing neuronal death post-TBI. After administering dexmedetomidine, the PSD95–NR2B–nNOS interaction was decreased efficiently, and they found dexmedetomidine could enhance cognitive and motor recovery following TBI [9]. Another study showed that dexmedetomidine can alleviate cerebral ischemic reperfusion injury in rats by increasing the α2-adrenergic receptor and blocking JNK phosphorylation and the activation of caspase-3 [10]. Feng’s study showed that dexmedetomidine improves neurological outcomes in mice and reduces neuronal death by protecting against neural autophagy and neuroinflammation. The neuroprotective capacity of DEX is partly dependent on the ROS/nuclear factor erythroid 2-related factor 2 signaling pathway [11].

There was a retrospective study in which dexmedetomidine was found to have a preventive effect on paroxysmal sympathetic hyperactivity in patients undergoing surgery for severe traumatic brain injury [22]. The length of the NICU stay and the hospital stay was shorter in the dexmedetomidine group than in the control group. But the difference was not significant, which was similar with our results.

Several studies found that the EEG theta/beta ratio (TBR) is associated with anxiety and executive control [23,24,25]. The negative relationship between the theta/beta ratio and trait attentional control was confirmed [23]. Angelidis et al. found that participants with a high TBR directed attention towards mildly threatening and avoided highly threatening pictures [24]. van Son D found that a high TBR was related to a low threat-bias in low trait-anxious people [25]. In this study, we found that dexmedetomidine could significantly increase the TBR. Midazolam could increase the TBR, but the increase was not significant. Patients in our study suffered from brain injury and were sedated. We could not get the information about anxiety or attentional control. After the patient’s consciousness improves, they may behave differently. This effect of dexmedetomidine needs to be validated in conscious patients.

The DAR and DTABR were reported to be independent predictors for clinical outcome [19]. A higher DAR and a higher DTABR were associated with a high mortality. In this study, the DTABR was significantly increased in both groups. But the DAR on both sides was just significantly increased in the midazolam group. Dexmedetomidine might be better than midazolam.

### 3.4. Limitations

There were some limitations in this study. First, this is a single-center clinical trial, and the sample was small. Second, patients in this study were undergoing craniotomy for three different reasons: brain trauma, cerebral hemorrhages, and cerebral infarctions. More than half of the patients underwent a craniotomy for a cerebral hemorrhage. Dexmedetomidine might improve some kind of patients’ outcome, however, there was no difference in results due to the confounding effect of including patients. Third, this study mainly observed clinical outcomes, and did not detect the function of nerve cells. We did not collect data to evaluate neurological function. Fourth, we just followed up the 28-day mortality. It is not clear about the effect of these two drugs on the long-term prognosis. Finally, GCS scores in the Dex group were higher than that in the Mid group, although there was no significant difference.

## 4. Conclusions

Compared with midazolam, dexmedetomidine did not lead to a lower 28-day mortality or better qEEG results in patients with brain injuries. The length of the ICU stay, the length of the hospital stay, and complications within 28 days were similar between the two groups.

## Figures and Tables

**Table 1:** Demographic and clinical characteristics of the Patients at Baseline.

Characteristics	Dexmedetomidine Group	Midazolam Group	F/χ^2^	*p*
Age-years	52.2 ± 13.5	51.8 ± 12.8	0.050	0.824
Male sex-no./total no. (%)	52/77 (67.5%)	52/74 (70.3%)	0.132 *	0.716
Weight-kg	70.8 ± 14.8	69.7 ± 13.5	0.216	0.643
Height-cm	167.8 ± 6.8	167.4 ± 6.9	0.109	0.741
BMI-kg/m^2^	25.0 ± 4.0	24.7 ± 3.7	0.173	0.678
Admission type-no./total no. (%)			1.907 *	0.385
Brain trauma	26/77 (33.8%)	31/74 (41.9%)		
Intracerebral hemorrhage	50/77 (64.9)	43/74 (58.1%)		
Cerebral infarction	1/77 (1.3%)	0		
APACHE II score	15.2 ± 5.7	15.7 ± 4.7	0.430	0.513
Glasgow score	6.5 ± 3.1	5.7 ± 2.3	3.730	0.055
RASS score	−3.8 ± 0.5	−3.9 ± 0.4	2.381	0.125
CPOT score	0.05 ± 0.28	0.04 ± 0.20	0.367	0.772
Propfol - no./total no. (%)	12/77 (15.6%)	4/74 (5.4%)	4.127 *	0.062
Mechanical ventilation-no./total no. (%)	74/77 (96.1%)	74/74 (100%)	2.942 *	0.086
Vasoactive drug-no./total no. (%)			0.001^*^	0.974
Vasoconstrictor	22/77 (28.6%)	23/74 (31.1%)		
Vasodilator	31/77 (40.3%)	32/74 (43.2%)		
None	24/77 (31.2%)	19/74 (25.7%)		
Chronic diseases-no./total no. (%)			3.793 *	0.580
Hypertension	37/77 (48.1%)	29/74 (39.2%)		
Diabetes	5/77 (6.5%)	3/74 (4.1%)		
Coronary heart disease	5/77 (6.5%)	3/74 (4.1%)		
Chronis kedney disease	3/77 (3.9%)	2/74 (2.7%)		
COPD	1/77 (1.3%)	0		
Stroke	4/77 (5.2%)	0		

BMI: Body mass index. APACHE: Acute physiology and chronic health evaluation. RASS: Richmond Agitation–Sedation Scale. COPT: Critical care pain observation tool. COPD: Chronic obstructive pulmonary disease. * χ^2^ values, otherwise F values.

**Table 2:** Clinical outcomes.

Outcome	Dexmedetomidine Group	Midazolam Group	F/χ^2^	*p*
28-day mortality-no./total no. (%)	11/77 (14.3%)	18/74 (24.3%)	2.451 *	0.117
Length of stay-days				
In ICU	9.5 ± 7.7	11.9 ± 11.6	2.279	0.133
In hospital	23.5 ± 19.8	25.3 ± 18.2	0.338	0.562
Complication within 28 days-no./total no. (%)			5.766 *	0.450
Pneumonia	61/77 (79.2%)	68/74 (91.9%)		
Intracranial infection	1/77 (1.3%)	3/74 (4.1%)		
Bloodstream infection	2/77 (2.6%)	1/74 (1.4%)		
Cerebral hemorrhage	1/77 (1.3%)	3/74 (4.1%)		
Cerebral infarction	0	3/74 (4.1%)		
Acute kidney injury	1/77 (1.3%)	0		
Ventricular tachycardia	1/77 (1.3%)	1/74 (1.4%)		

ICU: Intensive care unit. * χ^2^ values, otherwise F values.

**Table 3:** qEEG on day 1.

Outcome	Dexmedetomidine Group (*n* = 58)	Midazolam Group (*n* = 57)	F/χ^2^	*p*
EEG amplitude (μV)				
Left high	16.3 ± 8.1	14.7 ± 8.0	1.149	0.286
Left low	9.9 ± 4.7	8.9 ± 4.5	1.444	0.232
Right high	16.6 ± 7.5	14.3 ± 8.0	2.521	0.115
Right low	10.1 ± 4.3	8.7 ± 4.6	2.833	0.095
CV (LH)	26.5 ± 9.5	25.7 ± 9.4	0.252	0.616
CV (LL)	24.4 ± 9.2	23.2 ± 8.3	0.494	0.484
CV (RH)	28.6 ± 19.6	26.4 ± 8.1	0.614	0.435
CV (RL)	26.0 ± 18.0	23.3 ± 7.4	1.098	0.297
Spikes-no./total no. (%)	9/58 (15.5%)	5/57 (8.8%)	1.223 *	0.269
Alpha variance ( L )	21.7 ± 9.5	24.1 ± 11.1	1.504	0.223
Alpha variance ( R )	21.4 ± 12.2	23.2 ± 12.6	0.560	0.456
Percentagy of bands on the Left(%)				
Delta	83.1 ± 9.4	82.6 ± 15.5	0.042	0.839
Theta	7.8 ± 5.0	6.5 ± 4.7	1.906	0.170
Alpha	5.3 ± 3.6	6.2 ± 5.4	1.179	0.280
Beta	3.9 ± 3.5	4.7 ± 7.2	0.575	0.450
Percentagy of bands on the Right(%)				
Delta	86.1 (76.3–91.2)	87.7 (79.1–92.7)	0.056	0.814
Theta	7.6 ± 5.1	5.9 ± 3.9	4.112	0.045
Alpha	4.2 (1.9–6.4)	4.7 (1.8–7.9)	2.106	0.150
Beta	3.7 ± 3.8	4.5 ± 5.7	0.751	0.388
Spectral Entropy	55.2 ± 8.3	55.4 ± 8.1	0.014	0.906

LH: left high. LL:left low. RH: right high. RL: right low. CV: Coefficient of variation of EEG amplitude. TBR: theta/beta ratio. DTABR: (delta + theta)/(alpha + beta) ratio. * χ^2^ values, otherwise F values.

**Table 4:** qEEG on day 3.

Outcome	Dexmedetomidine Group (*n* = 46)	Midazolam Group (*n* = 42)	F/χ^2^	*p*
EEG amplitude (μV)				
Left high	17.0 ± 8.7	14.8 ± 6.9	1.613	0.208
Left low	10.4 ± 5.1	9.2 ± 4.2	1.395	0.241
Right high	15.0 ± 7.7	14.4 ± 6.3	0.138	0.711
Right low	9.3 ± 4.6	8.9 ± 3.9	0.228	0.635
CV (LH)	27.1 ± 11.7	24.1 ± 9.6	1.644	0.203
CV (LL)	24.9 ± 11.0	21.7 ± 8.3	2.393	0.126
CV (RH)	27.0 ± 10.2	27.0 ± 9.0	0.000	0.998
CV (RL)	24.1 ± 8.3	23.4 ± 7.6	0.164	0.687
Spikes-no./total no. (%)	5/46 (10.9%)	4/42 (9.5%)	0.043 *	0.835
Alpha variance (L)	16.6 ± 10.0	17.1 ± 13.0	0.057	0.811
Alpha variance (R)	16.8 ± 11.1	15.7 ± 10.1	0.230	0.633
Percentagy of bands on the Left(%)				
Delta	81.3 ± 16.7	85.9 ± 14.8	1.818	0.181
Theta	8.4 (3.8–15.0)	5.0 (3.1–10.9)	5.859	0.018
Alpha	4.8 ± 5.1	3.9 ± 4.8	0.617	0.434
Beta	3.8 ± 5.6	3.4 ± 6.7	0.111	0.740
Percentagy of bands on the Right(%)				
Delta	83.0 ± 15.3	87.0 ± 11.3	1.894	0.172
Theta	8.5 ± 4.7	6.2 ± 4.2	5.540	0.021
Alpha	4.3 ± 5.1	3.5 ± 3.2	0.751	0.389
Beta	4.2 ± 7.9	3.3 ± 6.0	0.384	0.537
Spectral Entropy	54.4 ± 7.7	51.9 ± 9.0	2.034	0.157

CV: Coefficient of variation of EEG amplitude. LH: left high. LL: left low. RH: right high. RL: right high. L: left. R: right. * χ^2^ values, otherwise F values.

**Table 5:** TBR, DAR, and DTABR.

Outcome		Dexmedetomidine Group (*n* = 46)	Midazolam Group (*n* = 42)	F	*p*
Left	TBR(Day1)	2.3 (1.3–4.7)	2.0 (1.0–2.6)	0.002	0.960
	TBR(Day3)	3.6 (1.8–5.8)	3.2 (2.0–6.0)	0.916	0.341
	*p*	0.000	0.057		
	DAR(Day1)	19.0 (9.5–35.0)	19.5 (10.1–35.8)	0.087	0.768
	DAR(Day3)	27.2 (14.2–57.4)	37.3 (19.3–75.7)	2.541	0.115
	*p*	0.070	0.006		
	DTABR(Day1)	13.4 (6.0–20.9)	11.9 (7.2–23.5)	0.057	0.811
	DTABR(Day3)	18.1 (8.7–18.1)	23.2 (13.4–47.1)	2.324	0.131
	*p*	0.000	0.019		
Right	TBR(Day1)	2.4 (1.2–6.4)	1.6 (0.9–3.7)	0.536	0.465
	TBR(Day3)	3.9 (2.0–7.8)	3.0 (1.9–6.8)	0.671	0.415
	*p*	0.000	0.061		
	DAR(Day1)	20.4 (11.6–43.3)	18.9 (10.1–52.3)	0.729	0.396
	DAR(Day3)	35.1 (16.7–65.0)	39.8 (17.5–99.9)	1.593	0.211
	*t*	−2.893	−3.279		
	*p*	0.006	0.002		
	DTABR(Day1)	13.3 (7.3–29.4)	12.3 (6.4–30.6)	0.121	0.728
	DTABR(Day3)	23.7 (12.4–42.6)	26.5 (11.0–58.9)	1.829	0.181
	*p*	0.002	0.000		

TBR: theta/beta ratio. DAR: delta/alpha ratio. DTABR: (delta + theta)/(alpha + beta) ratio.

## Data Availability

It will be available from the corresponding author on reasonable request.
